# Highly composite polynomials and the maximum order of the divisor function in $\pmb {\mathbb {F}}_q[t]$

**DOI:** 10.1007/s11139-020-00299-2

**Published:** 2020-08-03

**Authors:** Ardavan Afshar

**Affiliations:** Department of Mathematics, University College London, 25 Gordon Street, London, England

**Keywords:** Highly composite numbers, Divisor function, Arithmetic of polynomials over finite fields, Primary 11T55, Secondary 11N56

## Abstract

We investigate the analogues, in $\mathbb {F}_q[t]$, of highly composite numbers and the maximum order of the divisor function, as studied by Ramanujan. In particular, we determine a family of highly composite polynomials which is not too sparse, and we use it to compute the logarithm of the maximum of the divisor function at every degree up to an error of a constant, which is significantly smaller than in the case of the integers, even assuming the Riemann Hypothesis.

## Introduction

In [4], Ramanujan investigated the divisor function *d*(*n*), the number of divisors of *n*. Being interested in the maximum order of *d*(*n*), he defined highly composite integers *n* to be those for which $d(n) > d(n')$ for all $n > n'$, so that $D(N) := \max \{d(n) \ | \ n \leqslant N\}$ is given by $d(n')$ for the largest highly composite $n'\leqslant N$. He was able to compute $\log D(N)$ up to an error of at most $O(e^{-c\sqrt{\log \log N}}\log N)$ unconditionally and $O(\frac{\sqrt{\log N}}{(\log \log N)^3})$ assuming the Riemann Hypothesis. Ramanujan studied carefully the prime factorisation of the highly composite integers, and his results were improved by Alaoglu and Erdős in [1], who determined the exponent of each prime in the factorisation of a highly composite number up to an error of at most 1.

We consider the question of maximising the divisor function in the function field setting. Let $\mathbb {F}_q$ be a finite field, $\mathcal {M}= \{f \in \mathbb {F}_q[t] \text { monic}\}$, $\mathcal {M}_n = \{f \in \mathcal {M}: \deg f = n \}$, $\mathcal {I}= \{f \in \mathcal {M}\text { irreducible}\}$, $\mathcal {I}_n = \{f \in \mathcal {I}: \deg f = n\}$, and $\pi (n) = |\mathcal {I}(n)| = \frac{1}{n} \sum _{d | n} \mu (d) q^{n/d}$ where $\mu (d)$ is the Möbius function. For $f \in \mathcal {M}$, let $\tau (f)$ be the number of monic divisors of *f*, and observe that a generic polynomial *f* in $\mathcal {M}$ is of the form

1 $$\begin{aligned} f= \prod _{p \in \mathcal {I}} p^{a_p} \quad \text { with } \quad \deg f = \sum _{p \in \mathcal {I}} a_p \deg p \quad \text { and } \quad \tau (f) = \prod _{p \in \mathcal {I}} (1+a_p) \end{aligned}$$

where only finitely many $a_p$ are non-zero. We wish to understand the polynomials which maximise the function $\tau$ up to a given degree, defined thus:

### Definition 1.1

We call $f \in \mathcal {M}$ a *highly composite polynomial* of degree *n* if $\tau (f) = \max \{\tau (g) \ | \ g \in \bigcup _{m \leqslant n}\mathcal {M}_m\}$.

### Remark 1.2

Highly composite polynomials of a given degree are not necessarily unique. For example, all linear polynomials in $\mathcal {M}_1$ are highly composite polynomials of degree 1.

### Remark 1.3

There is (at least) one new highly composite polynomial at each degree. Indeed, let *f* be a highly composite polynomial of degree *n* and suppose otherwise, so that $\deg f = m < n$. Then pick some $g \in \mathcal {M}_{n-m}$, so that $fg \in \mathcal {M}_n$ but $\tau (fg) = \#\{d\in \mathcal {M}:d|fg\} \geqslant \#(\{d\in \mathcal {M}:d|f\} \cup \{fg\}) > \tau (f)$, which is a contradiction.

### Remark 1.4

If $f= \prod _{p \in \mathcal {I}} p^{a_p}$ is a highly composite polynomial, then $\deg p_i < \deg p_j$ implies $a_{p_i} \geqslant a_{p_j}$. Indeed, suppose otherwise and set $g = fp_i^{a_{p_j}-a_{p_i}}p_j^{a_{p_i}-a_{p_j}}$, so that $\tau (g) = \tau (f)$ but $\deg g = \deg f - (a_{p_j}-a_{p_i})(p_j - p_i) < \deg f$, which contradicts Remark 1.3.

### Remark 1.5

See “Appendix” for an illustrative table of highly composite polynomials in $\mathbb {F}_2[t]$.

We first investigate a family of highly composite polynomials $\{h(x)\}_{x>0}$ (following Ramanujan in [4]), which we define as follows:

### Definition 1.6

Let $x>0$. We say that $h=h(x) \in \mathcal {M}$ is an *x*-*superior highly composite* polynomial, or just *x*-SHC, if for all $f \in \mathcal {M}$ we have

2 $$\begin{aligned} \frac{\tau (h)}{q^{\deg h/x}} {\left\{ \begin{array}{ll} \geqslant \frac{\tau (f)}{q^{\deg f/x}} &{}\text { if } \deg h \geqslant \deg f ,\\ > \frac{\tau (f)}{q^{\deg f/x}} &{}\text { if } \deg h < \deg f, \end{array}\right. } \end{aligned}$$

and we say that *h* is an *x*-*semi-superior highly composite* polynomial, or *x*-SSHC, if for all $f \in \mathcal {M}$ we have

3 $$\begin{aligned} \frac{\tau (h)}{q^{\deg h/x}} \geqslant \frac{\tau (f)}{q^{\deg f/x}}. \end{aligned}$$

A polynomial which is *x*-SHC or *x*-SSHC for some $x>0$ is called *superior highly composite* or *semi-superior highly composite*, respectively.

### Remark 1.7

Clearly, if $h \in \mathcal {M}$ is *x*-SHC, then it is *x*-SSHC. Moreover, any polynomial *h* which is *x*-SSHC is highly composite, since if $f \in \mathcal {M}$ with $\deg f \leqslant \deg h$, then by Eq. (3) we have that

$$\begin{aligned} \tau (f) \leqslant \frac{\tau (h)}{q^{(\deg h - \deg f)/x}} \leqslant \tau (h). \end{aligned}$$

After defining a particular set for the parameter *x* of an *x*-SSHC,

### Definition 1.8

letting

$$\begin{aligned} S = S_q := \left\{ \frac{s \log q}{\log (1 + 1/r)} \ : \ s, r \geqslant 1 \right\} , \end{aligned}$$

we are able to determine the structure of the superior highly composite polynomials.

### Theorem 1

Let $$x > 0$$.

1. There is one, and only one, *x*-SHC polynomial, namely
  4 $$\begin{aligned} {\hat{h}} = {\hat{h}}(x) = \prod _{k\geqslant 1} \prod _{p \in \mathcal {I}_k} p^{a_k} \quad \mathrm{where} \quad a_k = a_k(x) = \left\lfloor \frac{1}{q^{k/x} - 1} \right\rfloor \end{aligned}$$
  Moreover, ${\hat{h}}$ is the unique highly composite polynomial of its degree.
2. If $x' < x''$ are two consecutive elements of *S*, then ${\hat{h}}(x) = {\hat{h}}(x')$ for all $x' \leqslant x < x''$. So, there is a one-to-one correspondence between *S* and the set of superior highly composite polynomials, given by $x \rightarrow {\hat{h}}(x)$,

and that of the semi-superior highly composite polynomials

### Theorem 2

Let $$x>0$$.

1. If $x \not \in S$, then there is only one *x*-SSHC polynomial, namely the polynomial ${\hat{h}}(x)$ defined in Eq. (4).
2. If $x = \frac{s \log q}{\log (1 + 1/r)} \in S$, then there are $2^{\pi (s)}$ *x*-SSHC polynomials of the form
  $$\begin{aligned} h(x) = \frac{{\hat{h}}(x)}{P_{i_1} \cdots P_{i_v}} \end{aligned}$$
  where ${\hat{h}}(x)$ is as in equation (4), $0 \leqslant v \leqslant \pi (s)$, $P_{i_1}, \ldots , P_{i_v} \in \mathcal {I}_s$ distinct, and $\deg h (x) = \deg {\hat{h}}(x) - vs$. Moreover, the unique polynomials *h* given by $v = \pi (s)$ and $v=0$ are two (distinct) consecutive superior highly composite polynomials.
3. If *h*(*x*) is *x*-SSHC and $g \in \mathcal {M}_{\deg h(x)}$ is a highly composite polynomial, then *g* is also *x*-SSHC.

This family of semi-superior highly composite polynomials is not too sparse, so we can use it to construct polynomials at every degree which make the divisor function close to its maximum. In particular, if we let $T(N) := \max \{\tau (f) \ | \ f \in \mathcal {M}_N \}$, then we are able to compute $\log T(N)$ within an error of at most $\log \frac{4}{3}$:

### Theorem 3

Let $x = \frac{s \log q}{\log (1 + 1/r)} \in S$, ${\hat{h}} = {\hat{h}}(x)$ and $a_k = a_k(x)$ be defined as in Eq. (4), and *h* be an *x*-SSHC polynomial of degree $\deg {\hat{h}}(x) - vs$ with $0 \leqslant v < \pi (s)$. Then, if $N = \deg h - u$ with $0 \leqslant u \leqslant s-1$, we have

$$\begin{aligned} \log T(N) = {\left\{ \begin{array}{ll} \log \tau (h) &{}\quad \mathrm{if } u=0 \\ \log \tau (h) - \epsilon (N) &{}\quad \mathrm{otherwise}, \end{array}\right. } \end{aligned}$$

where

$$\begin{aligned} \frac{u}{s}\log \left( 1 + \frac{1}{r}\right) \leqslant \epsilon (N) \leqslant \log \left( 1+\frac{1}{a_u}\right) . \end{aligned}$$

Moreover, the size of this range for $\epsilon (N)$ is at most $\log (1+\frac{1}{a_u(a_u + 2)}) \leqslant \log \frac{4}{3}$.

### Remark 1.9

From the final sentence of part 2 of Theorem 2, we know that the (distinct) superior highly composite polynomial ${\hat{h}}(x')$ immediately preceding ${\hat{h}}(x)$ has degree $\deg {\hat{h}}(x') = \deg {\hat{h}}(x) - \pi (s)s$. So, the form of *N* in Theorem 3 accounts for all integers between the degrees of these two consecutive superior highly composite polynomials. Therefore, for any $N \geqslant 1$, we can find $x>0$ so as to express *N* in the form presented in Theorem 3.

## Superior highly composite polynomials

We begin by showing, contingent on some auxiliary lemmas proven subsequently, that

### Proposition 2.1

For each $x>0$, the function $\frac{\tau (f)}{q^{\deg f /x}}$ is maximised over all $f \in \mathcal {M}$ by (at least one) $h = h(x) \in \mathcal {M}$. Moreover, if we write $h = \prod _{p \in \mathcal {I}} p^{a_p} = \prod _{p \in \mathcal {I}} p^{a_p(x)}$, we have that

1. If $x \not \in S$, then $a_p(x) = \lfloor \frac{1}{q^{\deg p/x} - 1} \rfloor$ for each $p \in \mathcal {I}$ and so *h* is unique.
2. Else, if $x = \frac{s \log q}{\log (1 + 1/r)} \in S$, so that $r = \frac{1}{q^{s/x} - 1}$, then
  $$\begin{aligned} a_p(x) = {\left\{ \begin{array}{ll} \left\lfloor \frac{1}{q^{\deg p/x} - 1} \right\rfloor &{}\quad \mathrm{if } \deg p \ne s, \\ r \text { or } r-1 &{}\quad \mathrm{if } \deg p = s\end{array}\right. } \end{aligned}$$
  and so there are $2^{\pi (s)}$ such polynomials *h*.

### Proof

From (1), we can write

$$\begin{aligned} \frac{\tau (f)}{q^{\deg f /x}} = \prod _{p \in \mathcal {I}} \frac{1+a_p}{q^{a_p \deg p / x}} = \exp \left( \sum _{p \in \mathcal {I}} \log (1+a_p) - \frac{a_ p \deg p \log q}{x}\right) \end{aligned}$$

so that to maximise $\frac{\tau (f)}{q^{\deg f /x}}$, for each $p \in \mathcal {I}$ we must maximise the quantity $\phi _{a_p} := \log (1+a_p) - \alpha a_p$ with $\alpha = \frac{\deg p \log q}{x}$.

If $x \not \in S$, then $\alpha$ cannot be written as $\log (1+\frac{1}{j})$ for any integer *j*, so by Lemma 2.3 we have that $\phi _{a_p}$ is maximised if and only if $a_p = \lfloor \frac{1}{e^\alpha - 1} \rfloor = \lfloor \frac{1}{q^{\deg p/x} - 1} \rfloor$.

Otherwise, if $x\in S$, then by Lemma 2.4 there is a unique pair (*s*, *r*) such that $x = \frac{s \log q}{\log (1 + 1/r)}$. Therefore, if $\deg p \ne s$, then $\alpha$ cannot be written as $\log (1+\frac{1}{j})$ for any integer *j*, so by Lemma 2.3 we have that $\phi _{a_p}$ is maximised if and only if $a_p = \lfloor \frac{1}{e^\alpha - 1} \rfloor = \lfloor \frac{1}{q^{\deg p/x} - 1} \rfloor$. Else, if $\deg p = s$, then $\alpha = \log (1+\frac{1}{r})$ and so by Lemma 2.3 we have that $\phi _{a_p}$ is maximised if and only if $a_p = r$ or $r-1$. $\square$

### Remark 2.2

Notice in both cases that $a_p(x)$ is zero for $\deg p > \frac{x \log 2}{\log q}$, so that the factorisation of *h* is in fact a finite product.

This leads us first to the proof of Theorem 1:

### Proof of Theorem 1

For $x>0$, let ${\hat{h}} = {\hat{h}}(x) = \prod _{k\geqslant 1} \prod _{p \in \mathcal {I}_k} p^{a_k}$ with $a_k(x) = \lfloor \frac{1}{q^{k/x} - 1} \rfloor$, and for $x = \frac{s \log q}{\log (1 + 1/r)} \in S$, let $E = E(x)$ be the set of polynomials defined in part 2 of Proposition 2.1.

1. If $x \not \in S$, then from part 1 of Proposition 2.1, we know that for all $f \in \mathcal {M}$, we have
  $$\begin{aligned} \frac{\tau ({\hat{h}})}{q^{\deg {\hat{h}}/x}} > \frac{\tau (f)}{q^{\deg f/x}} \end{aligned}$$
  and so ${\hat{h}}$ is the unique *x*-SHC. Moreover, if $\deg f \leqslant \deg {\hat{h}}$ then
  $$\begin{aligned} \tau ({\hat{h}}) > \tau (f)q^{(\deg {\hat{h}} - \deg f)/x} \geqslant \tau (f) \end{aligned}$$
  and so ${\hat{h}}$ is the unique highly composite polynomial of its degree. Otherwise, if $x = \frac{s \log q}{\log (1 + 1/r)} \in S$, so that $r = \frac{1}{q^{s/x} - 1}$, we observe that ${\hat{h}} = \prod _{p \in \mathcal {I}} p^{{\hat{a}}_p}$ with
  $$\begin{aligned} {\hat{a}}_p = {\hat{a}}_p(x) = \left\lfloor \frac{1}{q^{\deg p/x} - 1} \right\rfloor = {\left\{ \begin{array}{ll} \left\lfloor \frac{1}{q^{\deg p/x} - 1} \right\rfloor &{}\text { if } \deg p \ne s \\ r &{}\text { if } \deg p = s\end{array}\right. } \end{aligned}$$
  so that ${\hat{h}}(x) \in E$. Therefore, by part 2 of Proposition 2.1, we have that
  $$\begin{aligned} \frac{\tau ({\hat{h}})}{q^{\deg {\hat{h}}/x}} {\left\{ \begin{array}{ll} = \frac{\tau (f)}{q^{\deg f/x}} &{}\text { if } f \in E ,\\ > \frac{\tau (f)}{q^{\deg f/x}} &{}\text { if } f \not \in E , \end{array}\right. } \end{aligned}$$
  and that for all $f \in E\setminus \{{\hat{h}}\}$, we have $\deg f \leqslant \deg {\hat{h}} - s < \deg {\hat{h}}$. Therefore ${\hat{h}}$ is the unique *x*-SHC, and moreover, if $\deg f \leqslant \deg {\hat{h}}$ then
  $$\begin{aligned} \tau ({\hat{h}}) {\left\{ \begin{array}{ll} = \tau (f)q^{(\deg {\hat{h}} - \deg f)/x}> \tau (f) &{}\text { if } f \in E, \\ > \tau (f)q^{(\deg {\hat{h}} - \deg f)/x} \geqslant \tau (f) &{}\text { if } f \not \in E, \end{array}\right. } \end{aligned}$$
  and so ${\hat{h}}$ is the unique highly composite polynomial of its degree.
2. Let $x' < x''$ be two consecutive elements of *S*, and let ${\tilde{x}} = \min \{x > x' : {\hat{h}}(x) \ne {\hat{h}}(x') \}$. There are some ${\tilde{k}}$ such that $a_{{\tilde{k}}}({\tilde{x}}) = m > a_{{\tilde{k}}}(x')$. Therefore we must have that
  $$\begin{aligned} x'< \frac{{\tilde{k}} \log q}{\log \left( 1 + \frac{1}{m}\right) } \leqslant {\tilde{x}} < \frac{{\tilde{k}} \log q}{\log \left( 1 + \frac{1}{1+m}\right) } \end{aligned}$$
  and by the minimality of ${\tilde{x}}$ we must have that
  $$\begin{aligned} {\tilde{x}} = \frac{{\tilde{k}} \log q}{\log \left( 1 + \frac{1}{m}\right) } \in S. \end{aligned}$$
  So, by the minimality of ${\tilde{x}}$ and the definition of $x''$, we conclude that ${\tilde{x}} = x''$, and therefore that ${\hat{h}}(x) = {\hat{h}}(x')$ for all $x' \leqslant x < x''$. It follows that there is a one-to-one correspondence between *S* and the set of superior highly composite polynomials, given by $x \rightarrow {\hat{h}}(x)$.

$$\square $$

Then we move to the proof of Theorem 2:

### Proof of Theorem 2

For $x>0$, let ${\hat{h}} = {\hat{h}}(x) = \prod _{k\geqslant 1} \prod _{p \in \mathcal {I}_k} p^{a_k}$ with $a_k(x) = \lfloor \frac{1}{q^{k/x} - 1}\rfloor$, and for $x = \frac{s \log q}{\log (1 + 1/r)} \in S$, let $E = E(x)$ be the set of polynomials defined in part 2 of Proposition 2.1.

1. If $x \not \in S$, then the result follows from part 1 of Proposition 2.1.
2. If $x = \frac{s \log q}{\log (1 + 1/r)} \in S$, then from part 2 of Proposition 2.1, we have that the *x*-SSHC polynomials are precisely the $2^{\pi (s)}$ polynomials in the set *E*, which we can rewrite as
  $$\begin{aligned} E = \left\{ h(x) = \frac{{\hat{h}}(x)}{P_{i_1} \cdots P_{i_v}} : \ 0 \leqslant v \leqslant \pi (s) \text { and } P_{i_1}, \ldots , P_{i_v} \in \mathcal {I}_s \text { distinct}\right\} . \end{aligned}$$
  When $v = 0$, $h(x) = {\hat{h}}(x)$ is superior highly composite. When $v = \pi (s)$, $h(x) = \prod _{k\geqslant 1} \prod _{p \in \mathcal {I}_k} p^{{\tilde{a}}_k}$ where
  $$\begin{aligned} {\tilde{a}}_k = {\tilde{a}}_k(x) = {\left\{ \begin{array}{ll} a_k(x) &{}\text { if } k \ne s, \\ r - 1 &{}\text { if } k = s\end{array}\right. }. \end{aligned}$$
  Now, let $y = \max \{x' \in S: x' < x\}$ so that, by the definition of *x* and *y*, we have
  $$\begin{aligned} \frac{s \log q}{\log \left( 1 + \frac{1}{r-1}\right) } \leqslant y < x = \frac{s \log q}{\log \left( 1 + \frac{1}{r}\right) } \end{aligned}$$
  and so $a_k(y) = r-1$. When $k \ne s$, by Lemma 2.4, we cannot have that $x = \frac{k \log q}{\log (1 + 1/a_k(x))} \in S$, and so
  $$\begin{aligned} \frac{k \log q}{\log \left( 1 + \frac{1}{a_k(x)}\right) }< x < \frac{k \log q}{\log \left( 1 + \frac{1}{1 + a_k(x)}\right) }. \end{aligned}$$
  This means that, by the definition of *y*, we have for $k \ne s$ that
  $$\begin{aligned} \frac{k \log q}{\log \left( 1 + \frac{1}{a_k(x)}\right) } \leqslant y< x < \frac{k \log q}{\log \left( 1 + \frac{1}{1 + a_k(x)}\right) } \end{aligned}$$
  and so $a_k(y) = a_k(x)$. Therefore, we have that ${\hat{h}}(y) = h(x)$ and so, by part 2 of Theorem 1, *h*(*x*) is the (distinct) superior highly composite polynomial immediately preceding ${\hat{h}}(x)$.
3. Let *h*(*x*) be *x*-SSHC and $g \in \mathcal {M}_{\deg h(x)}$ be a highly composite polynomial. If $x \not \in S$, then by part 1 of Theorem 2, $h(x)={\hat{h}}(x)$, and by part 1 of Theorem 1, ${\hat{h}}(x)$ is the unique highly composite polynomial of its degree, so $g = {\hat{h}}(x) = h(x)$. Else, if $x \in S$, then by part 2 of Proposition 2.1, we have that $h \in E$ and
  $$\begin{aligned} \frac{\tau (h)}{q^{\deg h/x}} {\left\{ \begin{array}{ll} = \frac{\tau (f)}{q^{\deg f/x}} &{}\text { if } f \in E, \\ > \frac{\tau (f)}{q^{\deg f/x}} &{}\text { if } f \not \in E. \end{array}\right. } \end{aligned}$$
  Therefore $\tau (g) = \tau (h)$ if, and only if, $g \in E$ which means that *g* is also *x*-SSHC.

$$\square $$

Finally we conclude by proving the auxiliary lemmas used in the proof of Proposition 2.1, namely

### Lemma 2.3

Let $\alpha > 0$ and consider the sequence $(\phi _n)_{n \geqslant 0}$ defined by $\phi _n = \log (1+n) - \alpha n$. We have that

1. If $\log (1+\frac{1}{j+1})< \alpha < \log (1+\frac{1}{j})$ for some integer *j*, then $\phi _{n}$ is maximised if and only if $n = j = \lfloor \frac{1}{e^\alpha - 1} \rfloor$.
2. Else, if $\alpha = \log (1+\frac{1}{j})$ for some integer *j*, then $\phi _{n}$ is maximised if and only if $n = j$ or $j-1$.

### Proof

Let $\Delta _n = \phi _n - \phi _{n-1} = \log (1 + \frac{1}{n}) - \alpha$ for $n \geqslant 1$. Then we have that

1. If $\log (1+\frac{1}{j+1})< \alpha < \log (1+\frac{1}{j})$, then $\Delta _n > 0$ when $n \leqslant j$, and $\Delta _n < 0$ when $n > j$.
2. Else, if $\alpha = \log (1+\frac{1}{j})$, then $\Delta _n > 0$ when $n < j$, $\Delta _j = 0$ and $\Delta _n < 0$ when $n > j$.

$$\square $$

and

### Lemma 2.4

Let $x \in S$. Then there is a unique pair (*s*, *r*) such that $x = \frac{s \log q}{\log (1 + 1/r)}$.

### Proof

Suppose otherwise, so that $x = \frac{s \log q}{\log (1 + 1/r)} = \frac{S \log q}{\log (1 + 1/R)}$ with (*r*, *s*) and (*R*, *S*) distinct. If $s = S$ then $r=R$, so it must be that $s \ne S$ and in particular we may assume without loss of generality that $S>s$ so that $\frac{S}{s} > 1$.

Now $$\frac{s \log q}{\log (1 + 1/r)} = \frac{S \log q}{\log (1 + 1/R)}$$ implies $$\left( 1+\frac{1}{R}\right) ^s = \left( 1+\frac{1}{r}\right) ^S$$ and therefore $$\frac{(R+1)^s}{R^s} = \frac{(r+1)^S}{r^S}$$. However, $$\frac{(R+1)^s}{R^s} $$ and $$ \frac{(r+1)^S}{r^s}$$ are irreducible fractions, so we must have that $$R^s = r^S $$ and $$(R+1)^s = (r+1)^S$$. So, $$(1+r)^{S/s} = 1 + R = 1 + r^{S/s}$$, but $$(1+x)^{\alpha } > 1 + x^\alpha $$ for $$x > 0$$ and $$\alpha > 1$$, which is a contradiction. $$\square $$

## The maximum value of the divisor function at each degree

Since the family of semi-superior highly composite polynomials is not too sparse, we can use it to construct polynomials at every degree which make the divisor function close to its maximum, and thus prove Theorem 3:

### Proof of Theorem 3

If $u=0$, then $N = \deg h$, and by Theorem 2, *h* is highly composite, so $T(N) = \tau (h)$. Otherwise, if $1 \leqslant u \leqslant s-1$, we have by Remark 1.4 that $a_u(x) \geqslant a_s(x) = r \geqslant 1$. So, if we pick $P \in \mathcal {I}_u$ and let $g = \frac{h}{P}$, then $g \in \mathcal {M}$ with $\deg g = \deg h - u = N$ and therefore

$$\begin{aligned} T(N) \geqslant \tau (g) = \tau (h)\frac{a_u}{1+a_u} = \frac{\tau (h)}{1 + \frac{1}{a_u}} . \end{aligned}$$

On the other hand, by Theorem 2, *h* is *x*-SSHC, and so for any $f \in \mathcal {M}_N$, we have

$$\begin{aligned} \tau (f) \leqslant \tau (h) q^{(\deg f - \deg h)/x} = \tau (h) q^{-u/x} \end{aligned}$$

and therefore

$$\begin{aligned} T(N) \leqslant \tau (h) q^{-u/x} = \tau (h) \left( 1 + \frac{1}{r}\right) ^\frac{-u}{s} . \end{aligned}$$

Overall, this gives us that

5 $$\begin{aligned} \log q^{u/x} = \frac{u}{s}\log \left( 1 + \frac{1}{r}\right) \leqslant \log \tau (h) - \log T(N) \leqslant \log \left( 1+\frac{1}{a_u}\right) . \end{aligned}$$

Now, since

$$\begin{aligned} a_u = \left\lfloor \frac{1}{q^{u/x} - 1} \right\rfloor \geqslant \frac{1}{q^{u/x} - 1} - 1, \end{aligned}$$

we have that

$$\begin{aligned} q^{u/x} \geqslant 1 + \frac{1}{1 + a_u} \end{aligned}$$

and so the size of the range in equation (5) is at most

$$\begin{aligned} \log \left( 1+\frac{1}{a_u}\right) - \log \left( 1+\frac{1}{1 + a_u}\right) = \log \left( 1+\frac{1}{a_u(a_u + 2)}\right) \leqslant \log \frac{4}{3} . \end{aligned}$$

$$\square $$

## Appendix: Table of highly composite polynomials in $\pmb {\mathbb {F}}_2[t]$

We conclude with a table of highly composite polynomials in $$\mathbb {F}_2[t]$$, in which SSHC polynomials are additionally marked with $$*$$ and SHC polynomials are additionally marked with $$**$$. We denote the monic irreducible polynomials in $$\mathbb {F}_2[t]$$ by $$P_1(t), P_2(t), \ldots $$ in ascending order of the value which they take on $$t=2$$ (so that, if $$\deg P_i > \deg P_j$$, then $$i > j$$), and we write $$f \in \mathcal {M}$$ in the form $$f=P_1^{a_1} P_2^{a_2} \cdots $$ in order to shorten the printing. We have listed the explicit values of $$P_1(t), \ldots , P_{14}(t)$$, which are all of the irreducible polynomials which appear in the factorisations of polynomials in our table of highly composite polynomials, in Table 1, along with the values which they take on $$t=2$$.

**Table 1:** Table of ordered monic irreducible polynomials in $\mathbb {F}_2[t]$

*i*	$P_i(t) \in \mathcal {I}$	$\deg P_i$	$P_i(2)$
1	*t*	1	2
2	$t + 1$	1	3
3	$t^2 + t + 1$	2	7
4	$t^3 + t + 1$	3	11
5	$t^3 + t^2 + 1$	3	13
6	$t^4 + t + 1$	4	19
7	$t^4 + t^3 + 1$	4	25
8	$t^4 + t^3 + t^2 + t + 1$	4	31
9	$t^5 + t^2 + 1$	5	37
10	$t^5 + t^3 + 1$	5	41
11	$t^5 + t^3 + t^2 + t + 1$	5	47
12	$t^5 + t^4 + t^2 + t + 1$	5	55
13	$t^5 + t^4 + t^3 + t + 1$	5	59
14	$t^5 + t^4 + t^3 + t^2 + 1$	5	61

The algorithm which we use to compute highly composite polynomials is an adaption of the algorithm used to compute highly composite numbers in [2]. Though we take $q=2$, it works in the same way for any $\mathbb {F}_q[t]$, and we give a brief description as follows.

We first define the set $\mathcal {M}^{(k)} \subseteq \mathcal {M}$ of polynomials whose prime factors are in $\{P_1, \ldots , P_k\}$, and we call $f \in \mathcal {M}^{(k)}$ a *k*-*highly composite polynomial* if $\tau (f) = \max \{\tau (g) \ | \ g \in \mathcal {M}^{(k)} \text { and } \deg g \leqslant \deg f\}$. Then we let $\text {HC}(k, n) \subseteq \mathcal {M}^{(k)}$ be the set of *k*-highly composite polynomials of degree exactly *n*, and make the following observations:

- If $f = P_1^{a_1} P_2^{a_2} \cdots P_{k-1}^{a_{k-1}} P_k^{a_k} \in \text {HC}(k, n)$, then $g = P_1^{a_1} P_2^{a_2} \cdots P_{k-1}^{a_{k-1}} \in \text {HC}(k-1, n - a_k \deg P_k)$. Otherwise, if we had some $h \in \text {HC}(k-1, n - a_k \deg P_k)$ with $\tau (h) > \tau (g)$ then we would have $\tau (h P_k^{a_k}) > \tau (f)$ even though $h P_k^{a_k} \in \mathcal {M}^{(k)}$ with $\deg h P_k^{a_k} = n$, which would be a contradiction.
- If $f \in \text {HC}(k, n)$, and $P_i^{a_i}P_j^{a_j}$ divides *f* with $\deg P_j > \deg P_i$, then $a_i \geqslant a_j$. The proof of this is identical to that presented in Remark 1.4.

**Table 2:** Table of highly composite polynomials in $\mathbb {F}_2[t]$

$f \in \mathcal {M}$	$\deg f$	$\tau (f)$
$^{*}P_{1}^{1}$	1	2
$^{*}P_{2}^{1}$	1	2
$$ ^{**}P_{1}^{1} P_{2}^{1} $$	2	4
$^{*}P_{1}^{2} P_{2}^{1}$	3	6
$^{*}P_{1}^{1} P_{2}^{2}$	3	6
$$ ^{**}P_{1}^{2} P_{2}^{2} $$	4	9
$P_{1}^{3} P_{2}^{2}$	5	12
$P_{1}^{2} P_{2}^{3}$	5	12
$P_{1}^{2} P_{2}^{1} P_{3}^{1}$	5	12
$P_{1}^{1} P_{2}^{2} P_{3}^{1}$	5	12
$$ ^{**}P_{1}^{2} P_{2}^{2} P_{3}^{1} $$	6	18
$^{*}P_{1}^{3} P_{2}^{2} P_{3}^{1}$	7	24
$^{*}P_{1}^{2} P_{2}^{3} P_{3}^{1}$	7	24
$$ ^{**}P_{1}^{3} P_{2}^{3} P_{3}^{1} $$	8	32
$P_{1}^{4} P_{2}^{3} P_{3}^{1}$	9	40
$P_{1}^{3} P_{2}^{4} P_{3}^{1}$	9	40
$P_{1}^{4} P_{2}^{4} P_{3}^{1}$	10	50
$^{*}P_{1}^{3} P_{2}^{3} P_{3}^{1} P_{4}^{1}$	11	64
$^{*}P_{1}^{3} P_{2}^{3} P_{3}^{1} P_{5}^{1}$	11	64
$P_{1}^{4} P_{2}^{3} P_{3}^{1} P_{4}^{1}$	12	80
$P_{1}^{3} P_{2}^{4} P_{3}^{1} P_{4}^{1}$	12	80
$P_{1}^{4} P_{2}^{3} P_{3}^{1} P_{5}^{1}$	12	80
$P_{1}^{3} P_{2}^{4} P_{3}^{1} P_{5}^{1}$	12	80
$P_{1}^{4} P_{2}^{4} P_{3}^{1} P_{4}^{1}$	13	100
$P_{1}^{4} P_{2}^{4} P_{3}^{1} P_{5}^{1}$	13	100
$$ ^{**}P_{1}^{3} P_{2}^{3} P_{3}^{1} P_{4}^{1} P_{5}^{1} $$	14	128
$^{*}P_{1}^{4} P_{2}^{3} P_{3}^{1} P_{4}^{1} P_{5}^{1}$	15	160
$^{*}P_{1}^{3} P_{2}^{4} P_{3}^{1} P_{4}^{1} P_{5}^{1}$	15	160
$$ ^{**}P_{1}^{4} P_{2}^{4} P_{3}^{1} P_{4}^{1} P_{5}^{1} $$	16	200
$P_{1}^{5} P_{2}^{4} P_{3}^{1} P_{4}^{1} P_{5}^{1}$	17	240
$P_{1}^{4} P_{2}^{5} P_{3}^{1} P_{4}^{1} P_{5}^{1}$	17	240
$P_{1}^{4} P_{2}^{3} P_{3}^{2} P_{4}^{1} P_{5}^{1}$	17	240
$P_{1}^{3} P_{2}^{4} P_{3}^{2} P_{4}^{1} P_{5}^{1}$	17	240
$$ ^{**}P_{1}^{4} P_{2}^{4} P_{3}^{2} P_{4}^{1} P_{5}^{1} $$	18	300
$^{*}P_{1}^{5} P_{2}^{4} P_{3}^{2} P_{4}^{1} P_{5}^{1}$	19	360
$^{*}P_{1}^{4} P_{2}^{5} P_{3}^{2} P_{4}^{1} P_{5}^{1}$	19	360
$$ ^{**}P_{1}^{5} P_{2}^{5} P_{3}^{2} P_{4}^{1} P_{5}^{1} $$	20	432
$P_{1}^{6} P_{2}^{5} P_{3}^{2} P_{4}^{1} P_{5}^{1}$	21	504
$P_{1}^{5} P_{2}^{6} P_{3}^{2} P_{4}^{1} P_{5}^{1}$	21	504
$P_{1}^{4} P_{2}^{4} P_{3}^{2} P_{4}^{1} P_{5}^{1} P_{6}^{1}$	22	600
$P_{1}^{4} P_{2}^{4} P_{3}^{2} P_{4}^{1} P_{5}^{1} P_{7}^{1}$	22	600
$P_{1}^{4} P_{2}^{4} P_{3}^{2} P_{4}^{1} P_{5}^{1} P_{8}^{1}$	22	600
$P_{1}^{5} P_{2}^{4} P_{3}^{2} P_{4}^{1} P_{5}^{1} P_{6}^{1}$	23	720
$P_{1}^{4} P_{2}^{5} P_{3}^{2} P_{4}^{1} P_{5}^{1} P_{6}^{1}$	23	720
$P_{1}^{5} P_{2}^{4} P_{3}^{2} P_{4}^{1} P_{5}^{1} P_{7}^{1}$	23	720
$P_{1}^{4} P_{2}^{5} P_{3}^{2} P_{4}^{1} P_{5}^{1} P_{7}^{1}$	23	720
$P_{1}^{5} P_{2}^{4} P_{3}^{2} P_{4}^{1} P_{5}^{1} P_{8}^{1}$	23	720
$P_{1}^{4} P_{2}^{5} P_{3}^{2} P_{4}^{1} P_{5}^{1} P_{8}^{1}$	23	720
$^{*}P_{1}^{5} P_{2}^{5} P_{3}^{2} P_{4}^{1} P_{5}^{1} P_{6}^{1}$	24	864
$^{*}P_{1}^{5} P_{2}^{5} P_{3}^{2} P_{4}^{1} P_{5}^{1} P_{7}^{1}$	24	864
$^{*}P_{1}^{5} P_{2}^{5} P_{3}^{2} P_{4}^{1} P_{5}^{1} P_{8}^{1}$	24	864
$P_{1}^{6} P_{2}^{5} P_{3}^{2} P_{4}^{1} P_{5}^{1} P_{6}^{1}$	25	1008
$P_{1}^{5} P_{2}^{6} P_{3}^{2} P_{4}^{1} P_{5}^{1} P_{6}^{1}$	25	1008
$P_{1}^{6} P_{2}^{5} P_{3}^{2} P_{4}^{1} P_{5}^{1} P_{7}^{1}$	25	1008
$P_{1}^{5} P_{2}^{6} P_{3}^{2} P_{4}^{1} P_{5}^{1} P_{7}^{1}$	25	1008
$P_{1}^{6} P_{2}^{5} P_{3}^{2} P_{4}^{1} P_{5}^{1} P_{8}^{1}$	25	1008
$P_{1}^{5} P_{2}^{6} P_{3}^{2} P_{4}^{1} P_{5}^{1} P_{8}^{1}$	25	1008
$P_{1}^{4} P_{2}^{4} P_{3}^{2} P_{4}^{1} P_{5}^{1} P_{6}^{1} P_{7}^{1}$	26	1200
$P_{1}^{4} P_{2}^{4} P_{3}^{2} P_{4}^{1} P_{5}^{1} P_{6}^{1} P_{8}^{1}$	26	1200
$P_{1}^{4} P_{2}^{4} P_{3}^{2} P_{4}^{1} P_{5}^{1} P_{7}^{1} P_{8}^{1}$	26	1200
$P_{1}^{5} P_{2}^{4} P_{3}^{2} P_{4}^{1} P_{5}^{1} P_{6}^{1} P_{7}^{1}$	27	1440
$P_{1}^{4} P_{2}^{5} P_{3}^{2} P_{4}^{1} P_{5}^{1} P_{6}^{1} P_{7}^{1}$	27	1440
$P_{1}^{5} P_{2}^{4} P_{3}^{2} P_{4}^{1} P_{5}^{1} P_{6}^{1} P_{8}^{1}$	27	1440
$P_{1}^{4} P_{2}^{5} P_{3}^{2} P_{4}^{1} P_{5}^{1} P_{6}^{1} P_{8}^{1}$	27	1440
$P_{1}^{5} P_{2}^{4} P_{3}^{2} P_{4}^{1} P_{5}^{1} P_{7}^{1} P_{8}^{1}$	27	1440
$P_{1}^{4} P_{2}^{5} P_{3}^{2} P_{4}^{1} P_{5}^{1} P_{7}^{1} P_{8}^{1}$	27	1440
$^{*}P_{1}^{5} P_{2}^{5} P_{3}^{2} P_{4}^{1} P_{5}^{1} P_{6}^{1} P_{7}^{1}$	28	1728
$^{*}P_{1}^{5} P_{2}^{5} P_{3}^{2} P_{4}^{1} P_{5}^{1} P_{6}^{1} P_{8}^{1}$	28	1728
$^{*}P_{1}^{5} P_{2}^{5} P_{3}^{2} P_{4}^{1} P_{5}^{1} P_{7}^{1} P_{8}^{1}$	28	1728
$P_{1}^{6} P_{2}^{5} P_{3}^{2} P_{4}^{1} P_{5}^{1} P_{6}^{1} P_{7}^{1}$	29	2016
$P_{1}^{5} P_{2}^{6} P_{3}^{2} P_{4}^{1} P_{5}^{1} P_{6}^{1} P_{7}^{1}$	29	2016
$P_{1}^{6} P_{2}^{5} P_{3}^{2} P_{4}^{1} P_{5}^{1} P_{6}^{1} P_{8}^{1}$	29	2016
$P_{1}^{5} P_{2}^{6} P_{3}^{2} P_{4}^{1} P_{5}^{1} P_{6}^{1} P_{8}^{1}$	29	2016
$P_{1}^{6} P_{2}^{5} P_{3}^{2} P_{4}^{1} P_{5}^{1} P_{7}^{1} P_{8}^{1}$	29	2016
$P_{1}^{5} P_{2}^{6} P_{3}^{2} P_{4}^{1} P_{5}^{1} P_{7}^{1} P_{8}^{1}$	29	2016
$P_{1}^{4} P_{2}^{4} P_{3}^{2} P_{4}^{1} P_{5}^{1} P_{6}^{1} P_{7}^{1} P_{8}^{1}$	30	2400
$P_{1}^{5} P_{2}^{4} P_{3}^{2} P_{4}^{1} P_{5}^{1} P_{6}^{1} P_{7}^{1} P_{8}^{1}$	31	2880
$P_{1}^{4} P_{2}^{5} P_{3}^{2} P_{4}^{1} P_{5}^{1} P_{6}^{1} P_{7}^{1} P_{8}^{1}$	31	2880
$$ ^{**}P_{1}^{5} P_{2}^{5} P_{3}^{2} P_{4}^{1} P_{5}^{1} P_{6}^{1} P_{7}^{1} P_{8}^{1} $$	32	3456
$^{*}P_{1}^{6} P_{2}^{5} P_{3}^{2} P_{4}^{1} P_{5}^{1} P_{6}^{1} P_{7}^{1} P_{8}^{1}$	33	4032
$^{*}P_{1}^{5} P_{2}^{6} P_{3}^{2} P_{4}^{1} P_{5}^{1} P_{6}^{1} P_{7}^{1} P_{8}^{1}$	33	4032
$$ ^{**}P_{1}^{6} P_{2}^{6} P_{3}^{2} P_{4}^{1} P_{5}^{1} P_{6}^{1} P_{7}^{1} P_{8}^{1} $$	34	4704
$P_{1}^{7} P_{2}^{6} P_{3}^{2} P_{4}^{1} P_{5}^{1} P_{6}^{1} P_{7}^{1} P_{8}^{1}$	35	5376
$P_{1}^{6} P_{2}^{7} P_{3}^{2} P_{4}^{1} P_{5}^{1} P_{6}^{1} P_{7}^{1} P_{8}^{1}$	35	5376
$P_{1}^{6} P_{2}^{5} P_{3}^{3} P_{4}^{1} P_{5}^{1} P_{6}^{1} P_{7}^{1} P_{8}^{1}$	35	5376
$P_{1}^{5} P_{2}^{6} P_{3}^{3} P_{4}^{1} P_{5}^{1} P_{6}^{1} P_{7}^{1} P_{8}^{1}$	35	5376
$$ ^{**}P_{1}^{6} P_{2}^{6} P_{3}^{3} P_{4}^{1} P_{5}^{1} P_{6}^{1} P_{7}^{1} P_{8}^{1} $$	36	6272
$P_{1}^{7} P_{2}^{6} P_{3}^{3} P_{4}^{1} P_{5}^{1} P_{6}^{1} P_{7}^{1} P_{8}^{1}$	37	7168
$P_{1}^{6} P_{2}^{7} P_{3}^{3} P_{4}^{1} P_{5}^{1} P_{6}^{1} P_{7}^{1} P_{8}^{1}$	37	7168
$P_{1}^{7} P_{2}^{7} P_{3}^{3} P_{4}^{1} P_{5}^{1} P_{6}^{1} P_{7}^{1} P_{8}^{1}$	38	8192
$P_{1}^{6} P_{2}^{6} P_{3}^{3} P_{4}^{2} P_{5}^{1} P_{6}^{1} P_{7}^{1} P_{8}^{1}$	39	9408
$P_{1}^{6} P_{2}^{6} P_{3}^{3} P_{4}^{1} P_{5}^{2} P_{6}^{1} P_{7}^{1} P_{8}^{1}$	39	9408
$P_{1}^{6} P_{2}^{6} P_{3}^{2} P_{4}^{1} P_{5}^{1} P_{6}^{1} P_{7}^{1} P_{8}^{1} P_{9}^{1}$	39	9408
$P_{1}^{6} P_{2}^{6} P_{3}^{2} P_{4}^{1} P_{5}^{1} P_{6}^{1} P_{7}^{1} P_{8}^{1} P_{10}^{1}$	39	9408
$P_{1}^{6} P_{2}^{6} P_{3}^{2} P_{4}^{1} P_{5}^{1} P_{6}^{1} P_{7}^{1} P_{8}^{1} P_{11}^{1}$	39	9408
$P_{1}^{6} P_{2}^{6} P_{3}^{2} P_{4}^{1} P_{5}^{1} P_{6}^{1} P_{7}^{1} P_{8}^{1} P_{12}^{1}$	39	9408
$P_{1}^{6} P_{2}^{6} P_{3}^{2} P_{4}^{1} P_{5}^{1} P_{6}^{1} P_{7}^{1} P_{8}^{1} P_{13}^{1}$	39	9408
$P_{1}^{6} P_{2}^{6} P_{3}^{2} P_{4}^{1} P_{5}^{1} P_{6}^{1} P_{7}^{1} P_{8}^{1} P_{14}^{1}$	39	9408

The first observation allows us to iteratively compute $\text {HC}(k, n)$, as long as we know $\text {HC}(k-1, m)$ for all $m \leqslant n$. In particular, for each $j \geqslant 0$ with $n-j \deg P_k \geqslant 0$, we pick any (one) $g_j \in \text {HC}(k-1, n - j \deg P_k)$, and determine which values of *j* maximise $\tau (g_j P_k^j)$. Once we have determined such a set $J = \{j_1, \ldots , j_r\}$ we can conclude that

$$\begin{aligned} \text {HC}(k, n) = \{f_j P_k^j : j \in J, \ f_j \in \text {HC}(k-1, n - j \deg P_k)\}. \end{aligned}$$

It is trivial to observe that the base case $\text {HC}(1, n) = \{P_1^n\}$ for all $n \geqslant 0$, and we proceed inductively from there.

The second observation allows to note that, if $\{P_1, \ldots , P_k\} = \cup _{a \leqslant b} \mathcal {I}_a$ for some $b \geqslant 1$, and $\deg P_1 \cdots P_k \geqslant n$, then the set $\text {HC}(k, n)$ is in fact the set of highly composite polynomials of degree *n*. Thus, once we have $\text {HC}(k, n)$, by taking *k* sufficiently large, we are able to compute the highly composite polynomials of degree *n* (Table 2).

### Remark A.1

In $\mathbb {F}_2[t]$, there are certain degrees at which there is a unique highly composite polynomial of that degree, but where that polynomial is neither SHC nor SSHC (see degrees 10, 30 and 38 in the table of highly composite polynomials, for example). We leave for further investigation the question of whether there are infinitely many degrees with this property.

## References

[1] Alaoglu L, Erdős P (1944). On highly composite and similar numbers. Trans. Am. Math. Soc..

[2] Kedlaya, K.S.: An algorithm for computing highly composite numbers. https://shreevatsa.github.io/site/assets/hcn/hcn-algorithm.pdf. Accessed 28 May 2020

[3] Ramanujan S (1915). Highly composite numbers. Proc. Lond. Math. Soc..

[4] Ramanujan S (1927). Collected Papers of Srinivasa Ramanujan.

